# ^225^Ac α-Pretargeted Radioimmunotherapy of Human Epidermal Growth Factor Receptor 2–Expressing Breast Cancer

**DOI:** 10.2967/jnumed.125.269601

**Published:** 2025-11

**Authors:** Sara S. Rinne, Daniela Burnes Vargas, Shin Seo, Darren Veach, Michael R. McDevitt, Brett A. Vaughn, Hong Xu, Hong-Fen Guo, Edward K. Fung, Elisa de Stanchina, Ileana C. Miranda, Steven M. Larson, Nai Kong V. Cheung, Sarah M. Cheal

**Affiliations:** 1 Molecular Imaging Innovations Institute, Department of Radiology, Weill Cornell Medicine, New York, New York;; 2 Department of Pediatrics, Memorial Sloan Kettering Cancer Center, New York, New York;; 3 Molecular Pharmacology Program, Memorial Sloan Kettering Cancer Center, New York, New York;; 4 Department of Radiology, Memorial Sloan Kettering Cancer Center, New York, New York;; 5 Department of Radiology, Weill Cornell Medicine, New York, New York;; 6 Department of Medical Physics, Weill Cornell Medicine, New York, New York;; 7 Antitumor Assessment Core Facility, Memorial Sloan Kettering Cancer Center, New York, New York; and; 8 Laboratory of Comparative Pathology, Memorial Sloan Kettering Cancer Center, Weill Cornell Medicine, and Rockefeller University, New York, New York

**Keywords:** pretargeted radioimmunotherapy, actinium-225, trastuzumab, breast cancer, targeted alpha therapy

## Abstract

Radioimmunotherapy using ^225^Ac, a highly cytotoxic α-particle emitter, has potential for treating advanced breast cancer, especially human epidermal growth factor receptor 2 (HER2)–positive cases. We use a pretargeted radioimmunotherapy (PRIT) approach consisting of a 3-step intravenous regimen (step 1: bispecific anti-HER2/anti-DOTA antibody; step 2: clearing agent; step 3: ^225^Ac-radiolabeled *Proteus* DOTA, or [^225^Ac]Ac-Pr). Our goal was to establish curative ^225^Ac-PRIT with high therapeutic indices. **Methods:** The impact of [^225^Ac]Ac-Pr specific activity was evaluated in the BT-474 breast xenograft model. We tested the effects of [^225^Ac]Ac-Pr dosing during PRIT on tumor-targeting efficiency and tissue biodistribution. Using a ^225^Ac-PRIT regimen consisting of a ratio of 1.19 nmol of bispecific antibody to 0.60–0.66 nmol of [^225^Ac]Ac-Pr, we evaluated therapy in the BT-474 model and a patient-derived xenograft model. BT-474-tumor–bearing mice were treated with 1 or 2 cycles of ^225^Ac-PRIT (37 kBq/cycle) separated by 1 wk. A dose escalation study was performed on the BT-474 model to establish an absorbed radiation dose of approximately 40 Gy (relative biological effectiveness [RBE], 5) as a nephrotoxic dose, as no such histologic findings were observed in prior studies at the 20.7-Gy (RBE, 5) renal dose level. **Results:** In the BT-474 model, 100% (20/20) achieved complete responses and histologic cure in 17 of 20 (85%) of the treated animals. One-cycle and 2-cycle treatments were equally effective. Treatments were well tolerated, with no chronic radiation toxicity documented during necropsy at 175 d. Dosimetry estimates (RBE, 5) per 37 kBq administered for tumors and kidneys were 210 and 3.5 Gy, respectively. In the patient-derived xenograft model, a single ^225^Ac-PRIT treatment led to 60% (3/5) complete response and prolonged survival (>93 d) versus no treatment (30 d; *P* = 0.0185). Lastly, a ^225^Ac-PRIT regimen was identified that induces severe chronic nephrotoxicity (41.4 Gy/592 kBq; RBE, 5). **Conclusion:** Safe and effective ^225^Ac-PRIT regimens were developed in 2 preclinical models of advanced HER2-positive human breast cancer with tumor cure without dose-limiting nephrotoxicity. This study establishes crucial preclinical dosimetry benchmarks for ^225^Ac-PRIT and provides a compelling rationale for its advancement into the clinic.

## Visual Abstract

**Figure:**
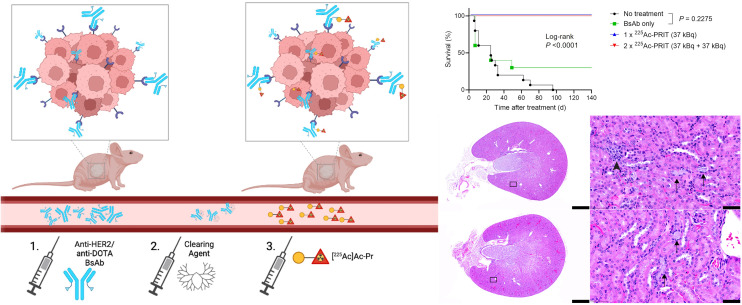

Advanced metastatic breast cancer has a poor prognosis, especially for highly aggressive triple-negative and human epidermal growth factor receptor 2 (HER2)–positive subtypes (1). *HER2* is an oncogene that is overexpressed in 15%–20% of breast cancers and is a clinically established therapeutic target (2,3). Although targeted therapies such as the HER2-targeted monoclonal antibody–drug conjugate trastuzumab–deruxtecan have improved outcomes, challenges remain because of treatment-related adverse events and tumor resistance to HER2 blockade in HER2-positive advanced-stage breast cancer (2).

Radioimmunotherapy, which combines therapeutic radionuclides with monoclonal antibodies to cancer-associated cell surface antigens, has therapeutic potential for many cancer types (4). HER2 is a promising target for radioimmunotherapy with the α-particle–emitting radionuclide ^225^Ac, as HER2-mediated internalization of the radioimmunoconjugate can potentially lead to increased retention of highly cytotoxic α-particles generated during ^225^Ac decay (half-life, 9.92 d) (5). It has been shown that the anti-HER2 humanized monoclonal IgG antibody trastuzumab radiolabeled with ^225^Ac is a potent therapeutic agent against HER2-positive breast cancer (6).

To enhance the therapeutic indices (TIs; tumor–to–normal-tissue absorbed dose ratios) of radioimmunotherapy using IgGs, a promising alternative strategy is to use a multistep or pretargeting approach (4). Various strategies have been explored for pretargeted radioimmunotherapy (PRIT), including with bispecific antibody (BsAb) (7). We established an anti-HER2 PRIT method consisting of administration of an anti-HER2/anti-DOTA hapten BsAb (an IgG single-chain variable fragment consisting of trastuzumab and the anti-DOTA[metal] complex single-chain variable fragment C825) followed by a clearing agent and ^177^Lu-labeled aminobenzylDOTA for delivery of therapeutic β-particle radiation (8). For ^225^Ac-therapy, we use *Proteus* DOTA (Pr), consisting of an empty DO3A-chelate for ^225^Ac, tethered via a short polyethylene glycol linker to a lutetium-complexed DOTA for picomolar C825 binding (as [^225^Ac]Ac-Pr) (9). We have also confirmed that Pr can be radiolabeled with its imaging surrogate, ^111^In (as [^111^In]In-Pr) (9). Notably, all radiolabeled forms of Pr contain stable lutetium (^175^Lu)-complexed aminobenzylDOTA as an affinity handle for direct binding to C825 (Fig. 1).

**FIGURE 1.**
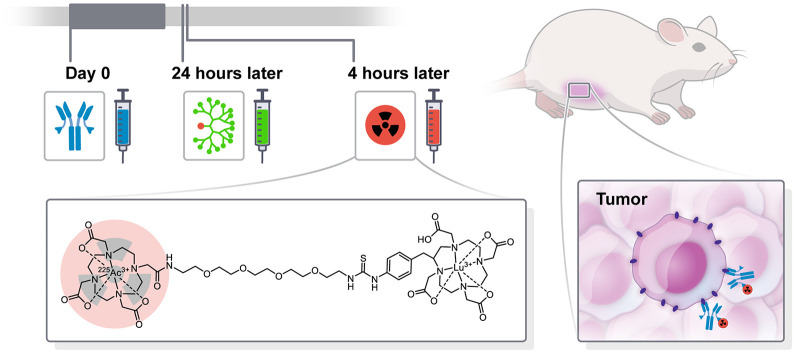
Schematic representation of 3-step HER2-targeted ^225^Ac-PRIT including chemical structure of [^225^Ac]Ac-Pr. For [^111^In]In-Pr, ^225^Ac is replaced with ^111^In.

This study aimed to establish high-TI ^225^Ac-PRIT for HER2-expressing breast cancer. Building on the work of Cheal et al. (9), we have significantly enhanced the specific activity of [^225^Ac]Ac-Pr (∼6-fold) (10). During the preliminary studies of HER2-pretargeted [^225^Ac]Ac-Pr, we observed moderate efficacy in the HER2-positive BT-474 breast cancer model (9). In the current study, our hypothesis was that we would see improved efficacy, including TI, based on the specific activity of the [^225^Ac]Ac-Pr. We took this opportunity to investigate how the administered mass of [^225^Ac]Ac-Pr influences tumor uptake and normal-tissue biodistribution in the BT-474 model during ^225^Ac-PRIT. We also investigated the kinetics of BsAb-pretargeted internalization of [^111^In]In-Pr as a surrogate for [^225^Ac]Ac-Pr, as internalization can significantly impact the potency of ^225^Ac targeted α-therapy (5). Dosimetry calculations were performed to establish dose–response and dose–toxicity relationships to correlate with antitumor efficacy and toxicities. The ^225^Ac-PRIT regimen was validated in a HER2-positive breast cancer patient–derived xenograft model.

## MATERIALS AND METHODS

General details regarding cell lines, PRIT reagents, and radiolabeling of Pr can be found in the supplemental materials (available at http://jnm.snmjournals.org).

### Mouse Models and PRIT Regimen

All animal procedures were performed in compliance with Memorial Sloan Kettering Cancer Center’s institutional Animal Care and Use Committee guidelines. The BT-474 model was established according to previously described procedures in female athymic nude mice (*nu/nu*) (9). Additional details are provided in the supplemental materials. A HER2-positive breast cancer patient–derived xenograft (M37 (12)) was established from fresh surgical specimens with Memorial Sloan Kettering Cancer Center Institutional Review Board approval in female BALB/c Rag2^−/−^IL-2Rγc^−/−^ mice.

The PRIT regimen was as follows for all biodistribution and therapy studies: the BsAb (0.25 mg, 1.19 nmol carrying 2.4 nmol of DOTA-[radio]hapten binding capacity) was administered at −28 h, followed by a clearing agent (25 µg, 2.76 nmol) at −4 h and [^225^Ac]Ac-Pr (as indicated below) at 0 h (Fig. 1) (11). Selection of this regimen was based on prior studies with ^177^Lu-PRIT in the BT-474 model (8).

### Cellular Binding and Internalization Assay

To assay the internalization kinetics of BsAb-pretargeted [^111^In]In-Pr, a method based on the work of Heskamp et al. (13) was used. We did not determine the internalization kinetics of BsAb-pretargeted [^225^Ac]Ac-Pr. Instead, following Kondo et al. (6), we assumed that substituting ^225^Ac for ^111^In would not alter these properties. Additionally, we presumed that the subcellular distribution of [^111^In]In-Pr and [^225^Ac]Ac-Pr in BT-474 cells would be similar, as suggested by Kondo et al. (14). More details are provided in the supplemental materials.

### Biodistribution and Dosimetry

In the BT-474 model, we compared the biodistribution profiles of ^225^Ac-PRIT with 2 different administered [^225^Ac]Ac-Pr molar doses. Groups of tumor-bearing mice were treated with BsAb and clearing agent and were injected with [^225^Ac]Ac-Pr at either 296 kBq (26.9 nmol; *n* = 6) or 37 kBq (0.60 nmol; *n* = 5). The higher dose was previously used to establish both efficacy and toxicity, whereas the lower dose was expected to be subsaturating in terms of absolute tumor uptake of Pr (9). After 24 h, the mice were euthanized for an ex vivo biodistribution assay. In an additional experiment, we performed serial biodistribution with ^225^Ac-PRIT in groups of BT-474 tumor–bearing mice (3–5/group; 37 kBq, 0.60 nmol) at 2, 24, 72, 192, and 240 h after injection to calculate dosimetry. A relative biological effectiveness (RBE) of 5 was used for α-radiation as suggested by Sgouros et al. (15). More details are provided in the supplemental materials.

### Radiopharmaceutical Therapy

Two separate studies were performed with BT-474 tumor–bearing mice (denoted as experiments 1 and 2). A single study was performed with M37 tumor–bearing mice (denoted as experiment 3). Supplemental Table 1 shows the starting tumor volumes and treatment group sizes (5–10/group) for all studies. During experiment 1, groups of BT-474 tumor–bearing mice were treated with 1 or 2 cycles of ^225^Ac-PRIT (cycle 1: 37 kBq, 0.64 nmol; cycle 2: 37 kBq, 0.66 nmol) separated by 1 wk. This regimen was based on prior studies showing no acute toxicity after ^225^Ac-PRIT (9). During each cycle, BsAb, clearing agent, and [^225^Ac]Ac-Pr were administered. During experiment 2, groups of BT-474 tumor–bearing mice were treated with 1 cycle of anti-HER2 BsAb only. Survival was defined as time to tumor doubling and included deaths from other causes. These study endpoints were based on prior studies with ^177^Lu-PRIT in the BT-474 model, which can have relatively slow growth kinetics (8). For experiment 1 only, surviving animals were submitted alive at 175 d to the Laboratory of Comparative Pathology of Memorial Sloan Kettering Cancer Center for pathologic analysis, hematology, and serum chemistry (the supplemental materials provide the anatomic and clinical pathology methods).

During experiment 3, groups of M37 tumor–bearing mice were treated with 1 cycle of ^225^Ac-PRIT (37 kBq, 0.64 nmol). A multicycle ^225^Ac-PRIT regimen was not attempted in this model. Treatment controls included [^225^Ac]Ac-Pr only or ^225^Ac-PRIT with control BsAb (antiglycoprotein A33 [GPA33]/anti-DOTA (16)) in place of anti-HER2 BsAb. Also, a biodistribution study (5 mice/group) was performed at 24 h after injection to confirm tumor targeting during ^225^Ac-PRIT. Survival was defined as the time to a tumor diameter of 10 mm and included deaths from other causes. No postmortem pathologic analysis, hematology, or serum chemistry was performed.

A complete response is defined as a tumor volume of 4.2 mm^3^ or smaller. A cure is defined as the absence of tumor cells at the implantation site, as confirmed by histological analysis.

### Dose Escalation Study to Evaluate Potential Nephrotoxicity

A dose escalation study was performed on a group of BT-474 tumor–bearing mice (*n* = 10) with treatment consisting of 2 cycles of ^225^Ac-PRIT separated by 1 wk (cycle 1: 296 kBq, 19.4 nmol; cycle 2: 296 kBq, 27.1 nmol). This treatment regimen delivered an estimated kidney-absorbed dose of 41.4 Gy. A nontreatment control group (*n* = 8) was included for comparison. Groups were monitored for 167 d after treatment initiation, and 6 randomly selected survivors from each group were submitted for necropsy and pathologic evaluation of toxicity. Antitumor effects were not monitored during this study.

### Data Analysis

Quantitative data were expressed as mean ± SD unless otherwise noted. Statistical and area-under-the curve analyses were performed using GraphPad Prism 9.3.1. Kaplan–Meier survival curves were analyzed with the Mantel–Cox test. Two-sided Student *t* tests were calculated, and a *P* value of less than 0.05 was considered to be statistically significant.

## RESULTS

### In Vitro Binding and Internalization of BsAb-Pretargeted [^111^In]In-Pr

**FIGURE 2.**
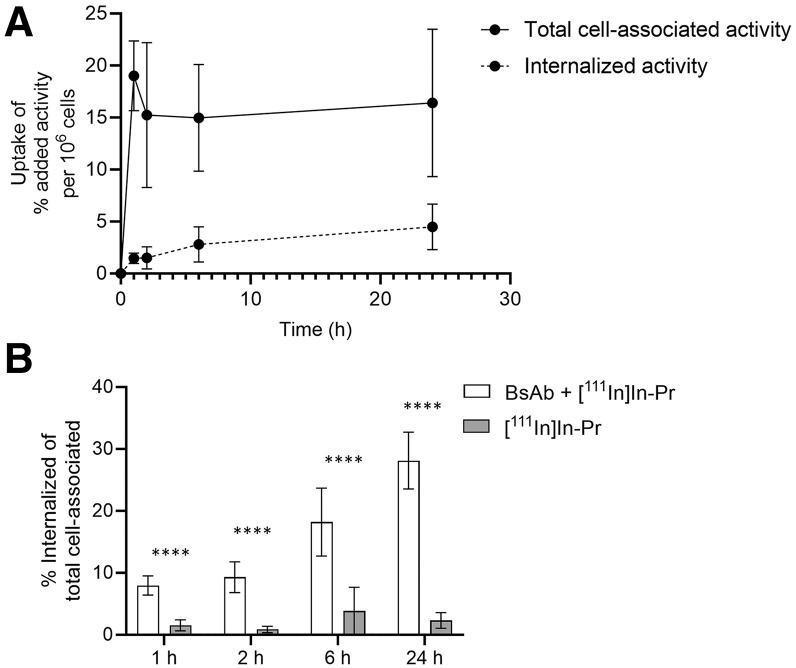
Internalization of anti-HER2 BsAb/[^111^In]In-Pr complex by HER2-positive BT-474 human breast cancer cells at 37°C. (A) Total bound and internalized activity of anti-HER2 BsAb + [^111^In]In-Pr. (B) Percentage internalized activity of total cell-bound for anti-HER2 BsAb + [^111^In]In-Pr or [^111^In]In-Pr control. ****P < 0.0001.

Cell binding and internalization of BsAb-pretargeted [^111^In]In-Pr were studied in vitro using BT-474 cell cultures (Fig. 2). After 1 h of 37°C incubation, rapid and efficient cell binding was observed (19.02% ± 3.36% added activity per 10^6^ cells) and [^111^In]In-Pr was mainly membrane-bound (8%–9% of total cell bound activity internalized). As shown in Figure 2A, peak cell binding was observed after 1 h and remained relatively constant up to 24 h (16.41% ± 7.09% added activity per 10^6^ cells). The internalized fraction gradually increased until 28% of the total cell-associated activity was internalized after 24 h (Fig. 2B). In the absence of BsAb, minimal binding and internalization were observed of [^111^In]In-Pr (*P* < 0.0001 for all time points), suggesting that [^111^In]In-Pr was internalized as the BsAb/[^111^In]In-Pr complex (Fig. 2B). The percentage internalized activity of total cell-associated activity was 19.3% based on area-under-the-curve analysis.

### Optimization of Administered [^225^Ac]Ac-Pr During ^225^Ac-PRIT

**FIGURE 3.**
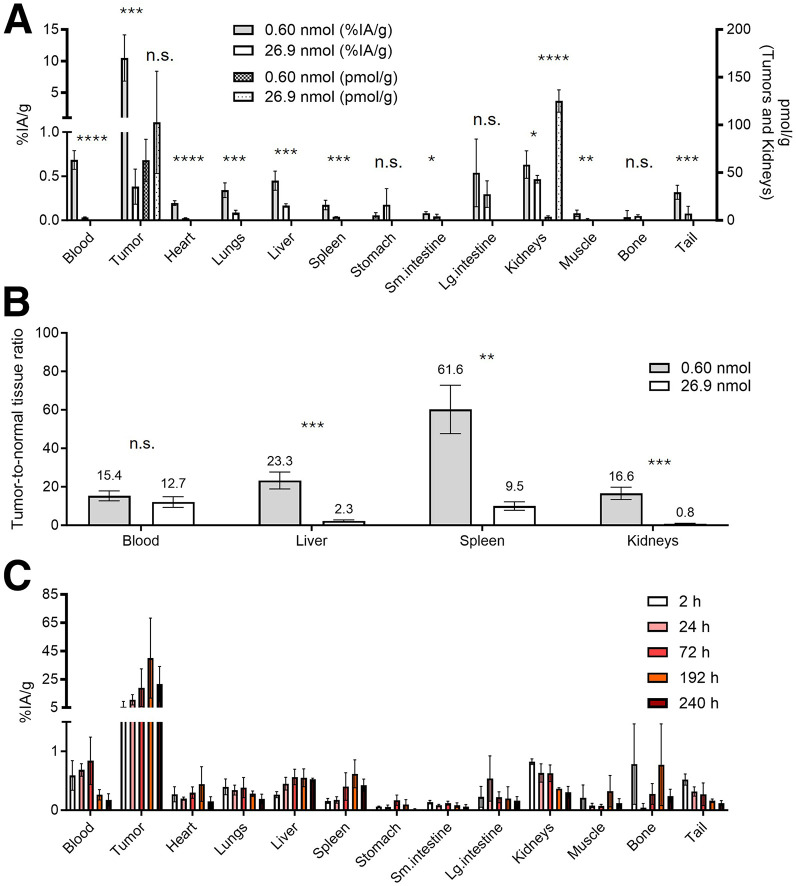
Influence of [^225^Ac]Ac-Pr mass dose on in vivo performance of HER2-targeted ^225^Ac-PRIT system. (A) Shown is ex vivo biodistribution assay 24 h after administration of [^225^Ac]Ac-Pr (37 kBq, 0.60 nmol; *n* = 5) or [^225^Ac]Ac-Pr (296 kBq, 26.9 nmol; *n* = 6) in groups of nude mice bearing subcutaneous BT-474 xenografts. Data are presented as %IA/g (left ordinate, all tissues) and pmol/g (right ordinate, tumors and kidneys only). Tabulated data and pmol/g for other tissues are provided in Supplemental Table 2. (B) Corresponding tumor–to–normal-tissue ratios. (C) Serial biodistribution data of optimized ^225^Ac-PRIT. Shown is ex vivo biodistribution assay after administration of [^225^Ac]Ac-Pr (37 kBq, 0.60 nmol) in groups of nude mice bearing subcutaneous BT-474 xenografts (3–5/group: 3 for 2 h, 5 for 24 and 72 h, and 3 for 192 and 240 h). Tabulated data are provided in Supplemental Table 3. *P < 0.05. **P < 0.01. ***P < 0.001. ****P < 0.0001. Lg = large; n.s. = no significant difference; Sm = small.

As a first step, we conducted a comparative biodistribution study on groups of BT-474 tumor–bearing mice to evaluate the effect of [^225^Ac]Ac-Pr mass during ^225^Ac-PRIT. These data are presented in Figures 3A and 3B and Supplemental Table 2. In summary, with the lower administered [^225^Ac]Ac-Pr mass (0.60 vs. 26.9 nmol), we observed significantly higher tumor uptake (10.48 ± 3.66 percentage injected activity [%IA]/g vs. 0.38 ± 0.20 %IA/g; *P* = 0.0001) at 24 h after injection. However, there was also significantly higher blood uptake (0.68 ± 0.11 %IA/g vs. 0.03 ± 0.01 %IA/g; *P* < 0.0001) and comparable kidney uptake (0.63 ± 0.16 %IA/g vs. 0.47 ± 0.04 %IA/g; *P* = 0.0322). Consequently, the 2 ^225^Ac-PRIT regimens resulted in similar tumor-to-blood ratios (15.4 vs. 12.7), but the low-mass dose regimen showed a significantly improved tumor-to-kidney uptake ratio (16.6 vs. 0.8), suggesting a more favorable TI for the kidney. The underlying mechanism is that during ^225^Ac-PRIT, the target has a saturable receptor density, and the amount of administered [^225^Ac]Ac-Pr mass can have a negative effect on tumor uptake due to saturation of the receptor sites at the targeted cancer cells with nonradioactive Pr.

### Serial Biodistribution and Dosimetry

Figure 3C and Supplemental Table 3 show the time-dependent biodistribution of ^225^Ac-PRIT (37 kBq, 0.60 nmol) in mice with BT-474 tumor xenografts. Time-integrated activities were calculated from these serial biodistribution data, and murine-specific internal dosimetry was estimated on the basis of total local absorption of α-emissions. Dosimetry for the tumors and kidneys for ^225^Ac-PRIT (296 kBq, 26.9 nmol) was extrapolated from ^225^Ac-PRIT (37 kBq, 0.60 nmol) by scaling the 24-h uptake data, assuming similar time–activity curve shapes. Similarly to Konijnenberg et al. (17), we postulate that the clearance kinetics of the [^225^Ac]Ac-Pr were unaffected by receptor saturation at the tumor. As shown in Figure 3A and Supplemental Table 2, saturation was not likely in the kidneys. As shown in Table 1, the estimated absorbed dose for BT-474 tumors was 33-fold higher during ^225^Ac-PRIT with [^225^Ac]Ac-Pr (37 kBq, 0.60 nmol, 5.670 Gy/kBq vs. 26.9 nmol, 0.170 Gy/kBq). The kidney-absorbed doses, however, were comparable (respectively: 0.60 nmol, 0.095 Gy/kBq; 26.9 nmol, 0.070 Gy/kBq). Additional dosimetry is provided in Supplemental Table 4.

**TABLE 1.** Dosimetry of ^225^Ac-PRIT in BT-474 Model

Parameter	[^225^Ac]Ac-Pr, 0.60 nmol	[^225^Ac]Ac-Pr, 26.9 nmol	Ratio
Tumor	5.670 Gy/kBq	0.170 Gy/kBq	33.4:1
Kidney	0.095 Gy/kBq	0.070 Gy/kBq	1.4:1
Kidney TI	60.0	2.5	

Dosimetry for ^225^Ac-PRIT (296 kBq, 26.9 nmol) was extrapolated from ^225^Ac-PRIT (37 kBq, 0.60 nmol) data (Fig. 3C; Supplemental Table 3).

### Radiopharmaceutical Therapy

**FIGURE 4.**
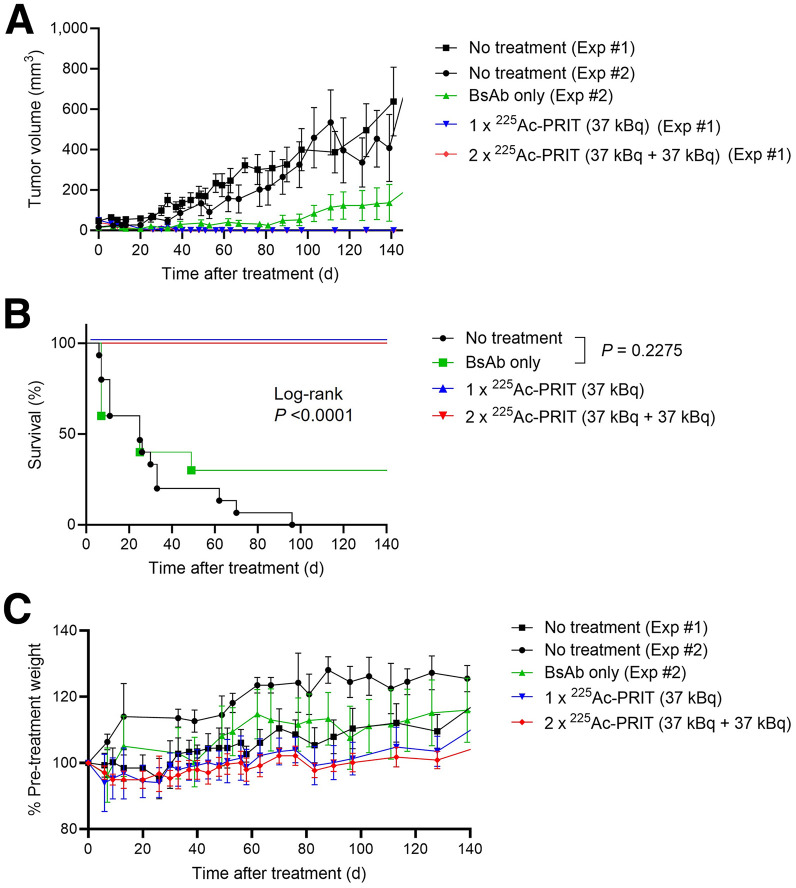
^225^Ac-PRIT treatment of BT-474 human breast cancer cell–derived xenograft. Data are shown as mean ± SE of mean. (A) Tumor growth curves after ^225^Ac-PRIT treatment. (B) Kaplan–Meier survival curves for pooled data (criterion: time for tumor to double in volume or death for any reason). (C) Normalized body weight changes after ^225^Ac-PRIT treatment. *n* = 5 for no treatment experiment 1, and *n* = 10 for all other treatment groups. Exp = experiment.

Treatment of BT-474 tumor–bearing mice with 1 or 2 cycles of ^225^Ac-PRIT (cycle 1: 37 kBq, 0.64 nmol; cycle 2: 37 kBq, 0.66 nmol) led to significant tumor regression, including complete responses (Fig. 4A). Furthermore, treatment with ^225^Ac-PRIT showed significantly enhanced survival over nontreated controls and anti-HER2 BsAb-only controls (log-rank, *P* < 0.0001) (Fig. 4B). The median survival was 25 d, 25 d, and not yet reached (>140 d) for nontreated, anti-HER2 BsAb-only, and ^225^Ac-PRIT, respectively. No significant difference in median survival was observed between nontreated and anti-HER2 BsAb-only controls (*P* = 0.2275). Both 1-cycle and 2-cycle treatments produced 100% (20/20) complete responses and histologic cure in 17 of 20 animals, or 85% of the treated animals. The therapy results are summarized in Supplemental Table 5.

For the M37 PDX model, a single ^225^Ac-PRIT cycle (37 kBq, 0.64 nmol) led to 60% (3/5) complete responses and prolonged survival (>93 d) compared with no treatment (30 d; *P* = 0.0185), [^225^Ac]Ac-Pr only (23 d; *P* = 0.0086), or GPA33 (nonspecific) ^225^Ac-PRIT (36 d; *P* = 0.0364). One mouse treated with ^225^Ac-PRIT could not be located starting at 30 d (tumor of 3.6 mm^3^ at 23 d). The therapy results are summarized in Supplemental Figure 1 and Supplemental Table 7. A biodistribution assay confirmed efficient tumor targeting during ^225^Ac-PRIT (Supplemental Table 6).

### Toxicity

All treatments in BT-474 tumor–bearing mice were well tolerated, with no acute toxicity. No BT-474 treatment groups showed average weight loss greater than 5% of the pretreatment baseline (Fig. 4C). For M37-treated groups, mild (∼10% change from baseline) but transient weight loss was observed (Supplemental Fig. 2). However, in the [^225^Ac]Ac-Pr–only and the GPA33 (nonspecific) ^225^Ac-PRIT control groups, 2 of 5 mice in each cohort were found deceased (cause of death unknown). For the [^225^Ac]Ac-Pr–only group, this occurred at 16 d despite weight losses of 8% and 10% from pretreatment baseline (tumor volumes at 8 d, 77.8 and 33.6 mm^3^, respectively). For the GPA33 (nonspecific) ^225^Ac-PRIT group, this occurred at 23 d despite weight losses of 9% and 4% from pretreatment baseline (tumor volumes at 16 d, 95.1 and 43.5 mm^3^, respectively).

**FIGURE 5.**
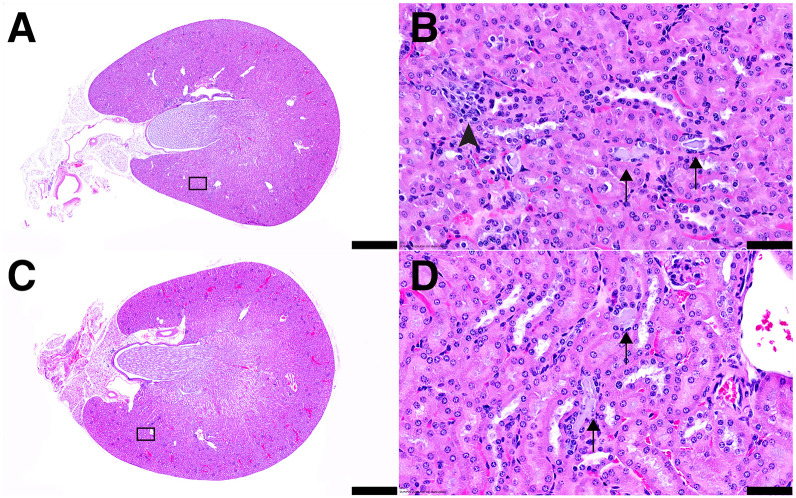
Representative histologic images of kidneys stained with hematoxylin and eosin. (A) Kidney from untreated control mouse showing normal shape and size (scale bar, 1 mm). (B) Boxed region from A showing minimal alterations, such as presence of amphophilic material within renal tubules (arrows) and interstitial inflammatory infiltrates (arrowhead) (scale bar, 50 µm). (C) Kidney from mouse at 175 d treated with 2 cycles of ^225^Ac-PRIT separated by 1 wk (cycle 1: 37 kBq, 0.64 nmol; cycle 2: 37 kBq, 0.66 nmol) showing normal shape and size (scale bar, 1 mm). Estimated kidney-absorbed dose is 7.0 Gy. (D) Boxed region from C showing minimal alterations, such as presence of amphophilic material within renal tubules (arrows) (scale bar, 50 µm).

From the BT-474 study, all ^225^Ac-PRIT–treated animals and a subset of untreated controls (5 untreated controls and 10 each of 1- and 2-cycle ^225^Ac-PRIT) were submitted for final necropsy at 175 d. A variety of histopathologic changes were noted; these are presented in Supplemental Table 8. We found no evidence of treatment-related radiotoxicity. A summary of the histologic scoring of renal degenerative tubulointerstitial changes is presented in Table 2. In Figure 5, representative histologic images of kidneys stained with hematoxylin and eosin are shown. No postmortem pathologic analysis was performed during the M37 study.

**TABLE 2.** Histologic Scoring of Renal Degenerative Tubulointerstitial Changes

Renal tubulointerstitial features	[^225^Ac]-IgG, 12.95 kBq*	No treatment	1 × ^225^Ac-PRIT, 37 kBq	2 × ^225^Ac-PRIT, 74 kBq	1 × ^225^Ac-PRIT, 296 kBq^†^	2 × ^225^Ac-PRIT, 592 kBq
Abnormal/reactive nuclear change	Moderate, focal karyorrhexis	Minimal (<1%), focal to multifocal karyomegaly and karyorrhexis	Minimal (<1%), focal to multifocal karyomegaly and karyorrhexis	Minimal (<5%), focal to multifocal karyomegaly and karyorrhexis	Mild, focal karyomegaly and karyorrhexis	Moderate (<30%), focal to multifocal karyomegaly and karyorrhexis
Cytoplasmic vacuolization (% of cells)	>50	<1	<1	<5	<5	<25
Tubulolysis with collapse (% of tubules)	>50	<1	<1	<5	<5	>60
Loss of brush border (% of tubules)	70	<1	<1	<5	<5	>60
Atrophy (% of tubules)	—	<1	<1	<5	<5	>60
Shrinkage or simplification (% of residual tubules)	50	<1	<1	<5	<5	>60
Interstitial inflammation/interstitial fibrosis	—/—	Minimal (<1%), multifocal lymphoplasmacytic infiltrates/not applicable	Mild (5%–25%), multifocal lymphoplasmacytic infiltrates/not applicable	Mild (5%–25%), focal lymphoplasmacytic infiltrates/minimal (<5%), multifocal	Minimal, lymphoplasmacytic interstitial inflammation	Mild (5%–25%), multifocal lymphoplasmacytic infiltrates/marked (>60%), multifocal
Medullary tubules	Normal	Minimal (<1%) tubular degeneration	Minimal (<1%) tubular basophilia	Minimal (<1%) tubular degeneration and basophilia	Normal	Minimal (<10%) tubular degeneration

* From Jaggi et al. (29).

† From Cheal et al. (9).

From the BT-474 study, hematology (Supplemental Table 9) and blood chemistry (Supplemental Table 10) were unremarkable in all animals of the different groups. Low creatinine kinase and alkaline phosphatase levels in various animals of different groups are likely artifactual given the lack of consistent morphologic changes in the tissues synthesizing or containing such enzymes. No hematology or serum chemistry was performed during the M37 study.

### Dose Escalation Study

Treatment with 2 doses of 296 kBq of ^225^Ac-PRIT was tolerated without weight loss (Supplemental Fig. 3). A single animal was found dead at 63 d (cause of death unknown), but otherwise there were no unscheduled mortalities.

During necropsy at 167 d, a high incidence of treatment-related renal histopathology was observed (Supplemental Table 11). Significant tubular injury with interstitial fibrosis was observed in 6 of 6 treated mice that underwent necropsy. A summary of the histologic scoring of renal degenerative tubulointerstitial changes is presented in Table 2. Representative histologic images of kidneys stained with hematoxylin and eosin taken from a treated mouse are shown in Supplemental Figure 4. The evidence of renal injury was also observed grossly as pale, small kidneys; decreased kidney weight (range, 0.198–0.378 g; control range, 0.330–0.451 g); and moderately increased blood urea nitrogen and creatinine on serum chemistry (Supplemental Table 12). Hematology was unremarkable in all treated animals (Supplemental Table 13).

## DISCUSSION

In this report, we document that when high-specific-activity ^225^Ac-PRIT (37 kBq/mouse) was administered in the BT-474 model, a tumor-absorbed dose of 210 Gy was curative and a kidney-absorbed dose of 3.5 Gy caused subclinical toxicity without observable changes in blood urea nitrogen or creatinine. In preclinical models of human advanced breast cancer, we achieved efficient tumor targeting combined with high TIs by administering sufficient amounts of both BsAb and [^225^Ac]Ac-Pr, with a ratio of 1.19 nmol of BsAb (2.4 nmol of DOTA-binding sites) to 0.60–0.66 nmol of [^225^Ac]Ac-Pr. In prior studies, it was necessary to give significantly higher administered [^225^Ac]Ac-Pr (296 kBq/mouse, 50.3 Gy; Table 2) to achieve less effective tumor control than in the current studies with high-specific-activity ^225^Ac-PRIT (37 kBq/mouse, 210 Gy; Table 2). These results show that when the specific activity of [^225^Ac]Ac-Pr is improved, we can achieve improved TI for kidney (from 2.5 to 60.0) while also significantly improving the tumor-absorbed dose 33-fold from 0.170 to 5.670 Gy/kBq (Table 2).

Here, we also explored a multicycle ^225^Ac-PRIT regimen. One-cycle and 2-cycle treatments were equally effective. During a 2-cycle treatment (total administered [^225^Ac]Ac-Pr, 74 kBq/mouse), we estimated an absorbed dose to tumor and kidney of 420 and 7.0 Gy, respectively, with mild renal toxicity observed. This was consistent with our prior studies, which showed mild renal toxicity in mice treated with 296 kBq (9); there, we estimated an absorbed dose to kidney of 20.7 Gy. During the dose escalation study with a cumulative therapeutic activity of 592 kBq/mouse, significant tubular injury with interstitial fibrosis was observed in all animals (kidney dose, 41.4 Gy). Therefore, we conclude that the maximum tolerated dose to kidney for ^225^Ac-PRIT falls between 20.7 and 41.4 Gy. These findings are consistent with prior work (Table 2).

Effective anti-HER2 ^225^Ac-PRIT may face challenges related to internalization and HER2 resistance. HER2 is known to internalize, and internalization of BsAb during PRIT may impair the intratumoral capture of radioligand, leading to ineffective tumor targeting. Additionally, the internalization kinetics may vary depending on whether the radioligand binds to the BsAb or whether it causes crosslinking of the BsAb on the cell membrane. Nevertheless, we achieved high TIs in blood (TI, 42) and kidney (TI, 60.0) while delivering 5.670 Gy/kBq to the tumor. These TIs are significantly higher than those previously reported during HER2 ^177^Lu-PRIT with the same BsAb in the same model (8) and HER2 ^225^Ac-PRIT on HER2-expressing human ovarian peritoneal xenografts (18). However, in the latter (18), these differences may be attributed to the properties of the xenograft model, the route of administration of BsAb and [^225^Ac]Ac-Pr, and variations in HER2 expression levels between the BT-474 and SKOV3 cell lines (19). For HER2 ^177^Lu-PRIT (8), differences may be due to the type of clearing agent, type of radioligand (aminobenzyl-DOTA was used to complex ^177^Lu for ^177^Lu-PRIT), and administered [^177^Lu]Lu-aminobenzylDOTA mass. Differences in radioligand type also raise the potential for variations in radioligand-mediated BsAb crosslinking at the membrane. It is anticipated that in both [^177^Lu]Lu-aminobenzylDOTA and [^225^Ac]Ac-Pr, the anti-DOTA C825 antibody will bind only to the Lu-aminobenzylDOTA moiety with high affinity. Therefore, these factors should be examined in greater detail to understand how to optimize TIs during HER2 PRIT.

Another potential concern is inefficient BsAb binding to tumor with intrinsic or acquired trastuzumab resistance. In such cases, it may be beneficial to use an alternative anti-HER2 antibody that targets an epitope different from trastuzumab (e.g., HT-19 (20)). These 2 possible mechanisms of resistance and approaches to overcoming them will require additional hypothesis-based experimental data to resolve the issue.

Numerous preclinical investigations of HER2-directed α-radioimmunotherapy in breast cancer have been reported, including with alternative antibody formats to IgGs such as single-domain antibodies and diabodies that are cleared primarily through the kidney (21). Renal retention after targeted radiopharmaceutical therapy can result in high radiation doses and potentially lead to end-stage renal disease (22). The normal-tissue dose limit for kidneys in radiopharmaceutical therapy (toxicity endpoint, end-stage renal disease; toxicity rate, 5%) is 23–26 Gy (∼36-Gy biologically effective dose) (23). Long-term follow-up studies of patients treated with ^225^Ac-radiopharmaceuticals have shown treatment-related kidney failure, but no dose–toxicity relationships were established (24,25). Our findings then are consistent with these current recommendations for kidney dose tolerances for ^225^Ac-radiopharmaceutical therapy. We have determined that kidney, and particularly renal proximal tubular cells, are the critical tissue for radiation toxicity for ^225^Ac-PRIT.

We demonstrated that ^225^Ac-PRIT was highly efficacious in both the BT-474 model and a HER2-overexpressing patient-derived xenograft model. However, the response rates varied significantly between the 2 models. The difference is unlikely to be due to reduced tumor uptake of [^225^Ac]Ac-Pr in the patient-derived xenograft model (Supplemental Table 6), as both models show comparable uptake at 24 h after injection. Instead, the intrinsic cellular radiosensitivity may differ. We acknowledge that BT-474 has been shown to be relatively radiosensitive to both low– and high–linear-energy-transfer radiation in comparison to other frequently studied HER2-overexpressing human cancer cell lines (26). Another contributing factor could be the potential differences in immune response due to the different mouse strains and the potential direct immunotherapeutic effects of the trastuzumab portion of the BsAb, which delays growth in the BT-474 model (Fig. 4) and the M37 model (12). Other factors influencing the therapeutic efficacy of targeted α-radiopharmaceutical therapy may include the subcutaneous tumor microenvironment and heterogeneity of dose distribution (27).

On the basis of the clinical history of PRIT and the high TIs reported, ^225^Ac-PRIT shows significant potential for clinical translation (7). However, the current 3-step approach, although useful as a test system, may pose barriers to clinical translation. To overcome these barriers, we are adapting the self-assembling and disassembling platform to target HER2. This platform has demonstrated the ability to achieve high TIs without requiring a clearing agent (28). Our strategy will be guided by ongoing clinical trials of self-assembling and disassembling PRIT (NCT05130255 and NCT05994157) and the recently completed trial of HER2-directed α-radioimmunotherapy (NCT04147819). PRIT has significant advantages with respect to TI that are likely to be needed for meaningful efficacy in this setting.

## CONCLUSION

This study illustrates the curative potential of ^225^Ac-PRIT as a treatment for highly aggressive subtypes of HER2-postive breast cancer. We determined that renal toxicity can be minimized by optimizing relative pharmacologic dosing of the BsAb carrier and radiohapten for safe and effective implementation of ^225^Ac-PRIT based on HER2 targeting.

## DISCLOSURE

This research was funded in part by the Hedvig Hricak Chair in Radiology (to Steven Larson), the Enid A. Haupt Chair (to Nai Kong Cheung), the Center for Targeted Radioimmunotherapy and Theranostics, the Ludwig Center for Cancer Immunotherapy of MSKCC (to Steven Larson), and Mr. William H. Goodwin and Mrs. Alice Goodwin and the Commonwealth Foundation for Cancer Research and the Experimental Therapeutics Center of MSKCC (to Steven Larson and Michael McDevitt). Steven Larson was also supported in part by P50-CA86438. This study also received support from R01-CA233896 (to Sarah Cheal). We also acknowledge P30-CA008748 for use of the Tri-Institutional Laboratory of Comparative Pathology, MSKCC, WCM, and the Rockefeller University, New York, NY; technical services provided by the MSKCC Small-Animal Imaging Core Facility and Laboratory of Comparative Pathology; as well as the Molecular Cytology Core Facility. MSKCC has filed for intraperitoneal protection for inventions related to the α-particle technology of which Michael McDevitt is an inventor. Michael McDevitt was a consultant for Actinium Pharmaceuticals, Regeneron, Progenics, Bridge Medicine, and General Electric. Both MSKCC and Nai Kong Cheung have financial interests in Y-mAbs Therapeutics, Inc., Abpro-Labs, and Lallemand-Biotec Pharmacon. Nai Kong Cheung reports receiving commercial research grants from Y-mAbs Therapeutics, Inc., and Abpro-Labs. Nai Kong Cheung was named as inventor on multiple patents filed by MSKCC, including those licensed to Y-mAbs Therapeutics, Inc., Lallemand-Biotec Pharmacon, and Abpro-Labs. Nai Kong Cheung is a scientific advisory board member for Eureka Therapeutics. Nai Kong Cheung, Steven Larson, and Sarah Cheal were named as inventors in the following patent applications relating to GPA33: SK2014-074, SK2015-091, SK2017-079, SK2018-045, SK2014-116, SK2016-052, and SK2018-068 filed by MSKCC. Steven Larson reports receiving commercial research grants from Genentech, Inc., WILEX AG, Telix Pharmaceuticals Limited, and Regeneron Pharmaceuticals, Inc.; holding ownership interest/equity in Elucida Oncology, Inc., and Y-mAbs Therapeutics, Inc., and holding stock in ImaginAb, Inc. Steven Larson is the inventor and owner of issued patents, both currently unlicensed and licensed by MSKCC to Samus Therapeutics, Inc., Elucida Oncology, Inc., and Y-mAbs Therapeutics, Inc. Steven Larson serves or has served as a consultant to Y-mAbs Therapeutics, Inc., Cynvec LLC, Eli Lilly & Co., Prescient Therapeutics Limited, Advanced Innovative Partners, LLC, Gerson Lehrman Group, Progenics Pharmaceuticals, Inc., and Janssen Pharmaceuticals, Inc. Steven Larson, Nai Kong Cheung, Darren Veach, and Sarah Cheal were named as inventors in PCT/US2021/039418 (THOR cell [tumor homing radio-emitting cell]). Sarah Cheal serves or has served as a consultant to Affibody AB and Primary Insight. No other potential conflict of interest relevant to this article was reported.

## Notes

KEY POINTS **QUESTION:** Can systemic HER2-targeted actinium radiopharmaceutical therapy be curative in mouse models of advanced human breast cancer? **PERTINENT FINDINGS:** In preclinical models of advanced human breast cancer, we have shown that treatment with optimized ^225^Ac-PRIT can result in a high probability of complete responses and durable tumor responses, including histologic cures. **IMPLICATIONS FOR PATIENT CARE:** We have identified a targeted α-radiopharmaceutical therapy with a high preclinical therapeutic window for treatment of HER2-expressing advanced breast cancer.

## References

[1] Swain SM Shastry M Hamilton E. Targeting HER2-positive breast cancer: advances and future directions. Nat Rev Drug Discov. 2023;22:101–126. 36344672 10.1038/s41573-022-00579-0 PMC9640784

[2] Marra A Chandarlapaty S Modi S. Management of patients with advanced-stage HER2-positive breast cancer: current evidence and future perspectives. Nat Rev Clin Oncol. 2024;21:185–202. 38191924 10.1038/s41571-023-00849-9 PMC12327481

[3] Wang J Xu B. Targeted therapeutic options and future perspectives for HER2-positive breast cancer. Signal Transduct Target Ther. 2019;4:34. 31637013 10.1038/s41392-019-0069-2 PMC6799843

[4] Larson SM Carrasquillo JA Cheung NK Press OW. Radioimmunotherapy of human tumours. Nat Rev Cancer. 2015;15:347–360. 25998714 10.1038/nrc3925 PMC4798425

[5] McDevitt MR Ma D Lai LT. Tumor therapy with targeted atomic nanogenerators. Science. 2001;294:1537–1540. 11711678 10.1126/science.1064126

[6] Kondo M Cai Z Chan C Forkan N Reilly RM. [^225^Ac]Ac- and [^111^In]In-DOTA-trastuzumab theranostic pair: cellular dosimetry and cytotoxicity in vitro and tumour and normal tissue uptake in vivo in NRG mice with HER2-positive human breast cancer xenografts. EJNMMI Radiopharm Chem. 2023;8:24. 37750937 10.1186/s41181-023-00208-0 PMC10522541

[7] Cheal SM Chung SK Vaughn BA Cheung N-KV Larson SM. Pretargeting: a path forward for radioimmunotherapy. J Nucl Med. 2022;63:1302–1315. 36215514 10.2967/jnumed.121.262186 PMC12079710

[8] Cheal SM Xu H Guo H-F. Theranostic pretargeted radioimmunotherapy of internalizing solid tumor antigens in human tumor xenografts in mice: curative treatment of HER2-positive breast carcinoma. Theranostics. 2018;8:5106–5125. 30429889 10.7150/thno.26585 PMC6217068

[9] Cheal SM McDevitt MR Santich BH. Alpha radioimmunotherapy using ^225^Ac-proteus-DOTA for solid tumors: safety at curative doses. Theranostics. 2020;10:11359–11375. 33052220 10.7150/thno.48810 PMC7546012

[10] Dacek MM Veach DR Cheal SM. Engineered cells as a test platform for radiohaptens in pretargeted imaging and radioimmunotherapy applications. Bioconjug Chem. 2021;32:649–654. 33819023 10.1021/acs.bioconjchem.0c00595 PMC8284561

[11] Cheal SM Patel M Yang G. An *N*-acetylgalactosamino dendron-clearing agent for high-therapeutic-index DOTA-hapten pretargeted radioimmunotherapy. Bioconjug Chem. 2020;31:501–506. 31891487 10.1021/acs.bioconjchem.9b00736 PMC7212493

[12] Lopez-Albaitero A Xu H Guo H. Overcoming resistance to HER2-targeted therapy with a novel HER2/CD3 bispecific antibody. Oncoimmunology. 2017;6:e1267891. 28405494 10.1080/2162402X.2016.1267891 PMC5384386

[13] Heskamp S Hernandez R Molkenboer-Kuenen JDM. Alpha- versus beta-emitting radionuclides for pretargeted radioimmunotherapy of carcinoembryonic antigen-expressing human colon cancer xenografts. J Nucl Med. 2017;58:926–933. 28232604 10.2967/jnumed.116.187021 PMC5450366

[14] Kondo M Cai Z Chan C Brown MK Reilly RM. Preclinical Comparison of [^111^In]In- and [^225^Ac]Ac-DOTA-trastuzumab IgG, F(ab′)_2_ and Fab′ for theranostic SPECT/CT imaging and alpha-particle radioimmunotherapy of HER2-positive human breast cancer. Mol Pharm. 2025;22:474–487. 39666273 10.1021/acs.molpharmaceut.4c01071 PMC11708818

[15] Sgouros G Roeske JC McDevitt MR.; SNM MIRD Committee. MIRD pamphlet no. 22 (abridged): radiobiology and dosimetry of alpha-particle emitters for targeted radionuclide therapy. J Nucl Med. 2010;51:311–328. 20080889 10.2967/jnumed.108.058651 PMC5680544

[16] Cheal SM Fung EK Patel M. Curative multicycle radioimmunotherapy monitored by quantitative SPECT/CT-based theranostics, using bispecific antibody pretargeting strategy in colorectal cancer. J Nucl Med. 2017;58:1735–1742. 28705917 10.2967/jnumed.117.193250 PMC5666642

[17] Konijnenberg MW Breeman WA de Blois E. Therapeutic application of CCK2R-targeting PP-F11: influence of particle range, activity and peptide amount. EJNMMI Res. 2014;4:47. 26116111 10.1186/s13550-014-0047-1 PMC4452684

[18] Chung SK Vargas DB Chandler CS. Efficacy of HER2-targeted intraperitoneal ^225^Ac alpha-pretargeted radioimmunotherapy for small-volume ovarian peritoneal carcinomatosis. J Nucl Med. 2023;64:1439–1445. 37348919 10.2967/jnumed.122.265095 PMC10478816

[19] Ram S Kim D Ober RJ Ward ES. The level of HER2 expression is a predictor of antibody-HER2 trafficking behavior in cancer cells. MAbs. 2014;6:1211–1219. 25517306 10.4161/mabs.29865 PMC4622696

[20] Le Joncour V Martins A Puhka M. A novel anti-HER2 antibody-drug conjugate XMT-1522 for HER2-positive breast and gastric cancers resistant to trastuzumab emtansine. Mol Cancer Ther. 2019;18:1721–1730. 31292166 10.1158/1535-7163.MCT-19-0207

[21] Altunay B Morgenroth A Beheshti M. HER2-directed antibodies, affibodies and nanobodies as drug-delivery vehicles in breast cancer with a specific focus on radioimmunotherapy and radioimmunoimaging. Eur J Nucl Med Mol Imaging. 2021;48:1371–1389. 33179151 10.1007/s00259-020-05094-1 PMC8113197

[22] Vegt E de Jong M Wetzels JF. Renal toxicity of radiolabeled peptides and antibody fragments: mechanisms, impact on radionuclide therapy, and strategies for prevention. J Nucl Med. 2010;51:1049–1058. 20554737 10.2967/jnumed.110.075101

[23] Wahl RL Sgouros G Iravani A. Normal-tissue tolerance to radiopharmaceutical therapies, the knowns and the unknowns. J Nucl Med. 2021;62(suppl 3):23S–35S. 34857619 10.2967/jnumed.121.262751 PMC12079726

[24] Kratochwil C Apostolidis L Rathke H. Dosing ^225^Ac-DOTATOC in patients with somatostatin-receptor-positive solid tumors: 5-year follow-up of hematological and renal toxicity. Eur J Nucl Med Mol Imaging. 2021;49:54–63. 34448031 10.1007/s00259-021-05474-1 PMC8712294

[25] Kairemo K Kgatle M Bruchertseifer F Morgernstern A Sathekge MM. Design of ^225^Ac-PSMA for targeted alpha therapy in prostate cancer. Ann Transl Med. 2024;12:67. 39118950 10.21037/atm-23-1842 PMC11304416

[26] Steffen AC Göstring L Tolmachev V Palm S Stenerlöw B Carlsson J. Differences in radiosensitivity between three HER2 overexpressing cell lines. Eur J Nucl Med Mol Imaging. 2008;35:1179–1191. 18193218 10.1007/s00259-007-0713-x

[27] Stenberg VY Tornes AJK Nilsen HR. Factors influencing the therapeutic efficacy of the PSMA targeting radioligand ^212^Pb-NG001. Cancers (Basel). 2022;14:2784. 35681766 10.3390/cancers14112784 PMC9179904

[28] Santich BH Cheal SM Ahmed M. A self-assembling and disassembling (SADA) bispecific antibody (BsAb) platform for curative two-step pretargeted radioimmunotherapy. Clin Cancer Res. 2021;27:532–541. 32958698 10.1158/1078-0432.CCR-20-2150 PMC7855367

[29] Jaggi JS Seshan SV McDevitt MR LaPerle K Sgouros G Scheinberg DA. Renal tubulointerstitial changes after internal irradiation with alpha-particle-emitting actinium daughters. J Am Soc Nephrol. 2005;16:2677–2689. 15987754 10.1681/ASN.2004110945

